# Mr. Hutchinson and Leprosy

**Published:** 1903-05-30

**Authors:** 


### MR. HUTCHINSON AND LEPROSY.

A Correspondent writes: "Sir,—The leading article entitled 'Mr. Hutchinson on Leprosy,' which appeared in your issue of April 18th last, contains a most important misstatement which should be corrected. It was stated in that article that Mr. Hutchinson's journey to India was undertaken 'mainly in response to the assertion that the natives were not fish eaters.' Considering the fact that fish forms one of the staple articles of food of large numbers of the natives of India, it is difficult to understand how this most extraordinary error could have arisen. Mr. Hutchinson certainly did not go to India to tilt against such a veritable windmill as that. When it is gravely stated that Mr. Hutchinson 'has brought back evidence that fish is largely eaten' in India, the statement is almost as ludicrous as it would be if a native of India were to say that he had discovered evidence to show that large quantities of coal were to be found in England.

"The main object of Mr. Hutchinson's visit to India was, as stated by himself, to explain his views on the subject of leprosy, views which he believed were not thoroughly understood by most medical men in this country; and also to invite discussion as to its etiology. The great stumbling-block to the fish theory has always been the fact that Brahmins, who not unfrequently contract leprosy, are nonfish eaters, and it was this aspect of the question that was chiefly to be discussed. At the meetings that were held in different parts of the country many native practitioners instanced cases of orthodox Brahmins who had become infected with leprosy. Mr. Hutchinson's invariable reply to these statements was to express his disbelief in the rigidity of caste restrictions by quoting a native saying to the effect that 'where there is no eye there is no caste.' Apart from the unsoundness of such an argument, Mr. Hutchinson thereby showed his complete ignorance of the manners and customs of the Brahmin community. He did not realise the fact that to the ordinary Brahmin the eating of fish would be as repulsive as cannibalism would be to the ordinary Englishman, and therefore there could be no temptation to the former to eat fish 'on the sly,' as Mr. Hutchinson would seem to think on those occasions when, with a humorous twinkle in his eye, he quoted the above-mentioned proverb. Mr. Hutchinson's visit to India has certainly done nothing to strengthen the arguments in favour of the fish theory."

[Our correspondent is to a great extent inaccurate in his assertions. Mr. Hutchinson's own version of the objects of his journey to India may be found in an article "furnished," evidently by himself, to the *Times*, and which appeared in that paper for April 11th. The Prince of Wales's Commission sent a committee to India, which reported that the fact "that a fair number of cases of leprosy exists among people who have never touched such food (*i.e.* fish) argues sufficiently strongly against the exclusive fish hypothesis." "Dissenting from this conclusion," says the *Times* article, "and believing that the statements of alleged facts to which the Commission had trusted were, in some respects, erroneous, Mr. Hutchinson wished to go over the ground again, and to examine these 'facts' more critically." His journey had no special reference to Brahmins, but was directed to ascertain whether the people who were alleged never to have used fish as food had, in reality, done so or not. He found, in a very large proportion of cases, that they had. At one asylum, for example, the native medical superintendent had prepared a schedule for Mr. Hutchinson, according to which nearly forty of the inmates alleged that they had never eaten fish. When cross-examined by Mr. Hutchinson himself, only one of them persisted in his denial; the others all admitting that they had eaten fish, and saying either that they had misunderstood the question or that the doctor had mistaken their answer. With regard to leprous Brahmins who had never eaten fish, if such there be, it must be remembered that Mr. Hutchinson fully admits the occurrence of what he calls "commensal communication" of the disease. Everybody knew, of course, that fish is largely eaten in India, but what Mr. Hutchinson ascertained was that it had been largely eaten by lepers who had been said never to have partaken of it at all.—ED. THE HOSPITAL.]

